# Anti-angiogenic effects of differentiation-inducing factor-1 involving VEGFR-2 expression inhibition independent of the Wnt/β-catenin signaling pathway

**DOI:** 10.1186/1476-4598-9-245

**Published:** 2010-09-16

**Authors:** Tatsuya Yoshihara, Fumi Takahashi-Yanaga, Fumie Shiraishi, Sachio Morimoto, Yutaka Watanabe, Masato Hirata, Sumio Hoka, Toshiyuki Sasaguri

**Affiliations:** 1 Department of Clinical Pharmacology, Faculty of Medical Sciences, Kyushu University, Fukuoka 812-8582, Japan; 2 Department of Anesthesia and Critical Care Medicine, Faculty of Medical Sciences, Kyushu University, Fukuoka 812-8582, Japan; 3 Department of Applied Chemistry, Faculty of Engineering, Ehime University, Matsuyama 790-8577, Japan; 4 Department of Molecular and Cellular Biochemistry, Faculty of Dental Sciences, Kyushu University, Fukuoka 812-8582, Japan

## Abstract

**Background:**

Differentiation-inducing factor-1 (DIF-1) is a putative morphogen that induces cell differentiation in *Dictyostelium discoideum*. DIF-1 inhibits proliferation of various mammalian tumor cells by suppressing the canonical Wnt/β-catenin signaling pathway. To assess the potential of a novel cancer chemotherapy based on the pharmacological effect of DIF-1, we investigated whether DIF-1 exhibits anti-angiogenic effects *in vitro *and *in vivo*.

**Results:**

DIF-1 not only inhibited the proliferation of human umbilical vein endothelial cells (HUVECs) by restricting cell cycle in the G_0_/G_1 _phase and degrading cyclin D1, but also inhibited the ability of HUVECs to form capillaries and migrate. Moreover, DIF-1 suppressed VEGF- and cancer cell-induced neovascularization in Matrigel plugs injected subcutaneously to murine flank. Subsequently, we attempted to identify the mechanism behind the anti-angiogenic effects of DIF-1. We showed that DIF-1 strongly decreased vascular endothelial growth factor receptor-2 (VEGFR-2) expression in HUVECs by inhibiting the promoter activity of human VEGFR-2 gene, though it was not caused by inhibition of the Wnt/β-catenin signaling pathway.

**Conclusion:**

These results suggested that DIF-1 inhibits angiogenesis both *in vitro *and *in vivo*, and reduction of VEGFR-2 expression is involved in the mechanism. A novel anti-cancer drug that inhibits neovascularization and tumor growth may be developed by successful elucidation of the target molecules for DIF-1 in the future.

## Background

Angiogenesis is a multi-step process essential for tumor growth and metastasis, which involves endothelial cell proliferation, migration and capillary formation [1-4]. Among many soluble and matrix-derived angiogenic growth factors and regulators of angiogenesis involved in neovascularization, vascular endothelial growth factor (VEGF) plays a crucial role in the proliferation, migration and survival of vascular endothelial cells [2,5-7].

The VEGF family consists of six members, VEGF-A, VEGF-B, VEGF-C, VEGF-D, VEGF-E and the placenta growth factor (PLGF) [4,7,8]. Among them, VEGF-A is known as the most important factor for many angiogenic processes. VEGF-A binds to two tyrosine kinase receptors, VEGFR-1 (Flt-1) and VEGFR-2 (KDR/Flk-1) [2,8-11]. Signaling through VEGFR-1 is related to embryonic angiogenesis and acts as a regulator of VEGFR-2 [7,8,12,13]. Although the affinity of VEGFR-2 for VEGF is lower than that of VEGFR-1, VEGFR-2 is more potent than VEGFR-1 in stimulating endothelial cell proliferation and migration [11,14]. VEGFR-2 expression is almost restricted to vascular endothelial cells and it has been reported that VEGFR-2 expression was markedly up-regulated during chronic inflammation, wound repair and tumor growth [5,15,16].

Differentiation-inducing factors (DIFs) were identified in *Dictyostelium discoideum *as morphogens required for stalk cell differentiation [17]. In the DIF family, DIF-1 (1-(3, 5-dichloro-2, 6-dihydroxy-4-methoxyphenyl)-1-hexanone) was the first to be identified. The actions of DIFs are not limited to *Dictyostelium *and they strongly inhibit the proliferation of human cells [18,19]. Previously, we reported that DIFs inhibited the Wnt/β-catenin signaling pathway via glycogen synthase kinase-3β (GSK-3β) activation, leading to cell cycle arrest at G_0_/G_1 _phase through suppression of cyclin D1 expression in various human tumor cells [20-23]. It is well known that the Wnt/β-catenin signaling pathway plays a number of key roles in embryonic development and maintenance of homeostasis in matured tissues. And also, this signaling pathway has been reported to play important roles in the proliferation and migration of endothelial cells, resulting in the promotion of angiogenesis [24-28].

In this study, we investigated the effect of DIF-1 on angiogenesis in *in vitro *and *in vivo *systems. We revealed that DIF-1 decreased the expression of VEGFR-2 in protein and mRNA levels via the suppression of the promoter activity by a Wnt/β-catenin signaling pathway-independent mechanism. Our results suggest that the suppression of VEGFR-2 expression could be one mechanism of the inhibition of angiogenesis induced by DIF-1 and that DIF-1 suppressed not only the Wnt/β-catenin signaling pathway but also neovascularization.

## Results

### DIF-1 inhibited HUVEC proliferation

**Figure 1:**
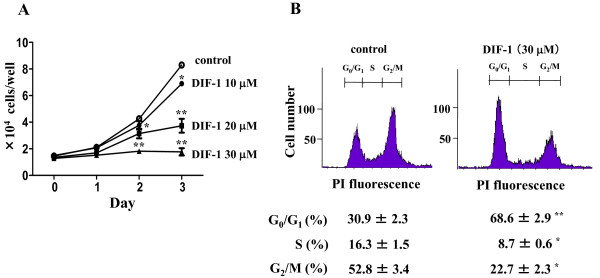
**DIF-1 inhibited HUVECs proliferation**. (A) Cell proliferation assay. HUVECs seeded on a 24-well plate (1.0×10^4 ^cells/well) were incubated with various amount of DIF-1. Cells were harvested by trypsin/EDTA treatment at the times indicated and enumerated. Values are mean ± SE of three independent experiments and statistically analyzed using a one-way ANOVA with Bonferroni post-hoc test. The asterisk indicates *P < 0.05 and **P < 0.01 versus control. (B) Flow cytometry. HUVECs were incubated with DIF-1 (30 μM) for 24 h and then harvested by the trypsin/EDTA treatment. Cells were stained with propidium iodide (PI) and fluorescence of nuclei was measured. The percentages of cell number in the cell cycle phases are also shown. The results are means ± SE of three independent experiments. The asterisk indicates *P < 0.01 and **P < 0.001 versus control.

DIF-1 exhibits powerful anti-proliferative effects in various mammalian cells [18-23] and we previously reported that DIF-3 induced cell cycle arrest by reducing cyclin D1 in HUVECs [20]. In this present study, we first examined whether DIF-1 also inhibited HUVEC proliferation. As shown in Figure 1A, DIF-1 strongly inhibited HUVEC proliferation in a dose-dependent manner. This anti-proliferative effect was unlikely to be caused by cytotoxicity, because the number of dead cells indicated by the trypan blue exclusion test was not increased by treatment with DIF-1 (data not shown). We next examined the effects of DIF-1 on cell cycle distribution using flow cytometry. As shown in Figure 1B, the cell population in the G_0_/G_1 _phase significantly increased and the population in S and G_2_/M phases decreased, indicating that DIF-1 induced G_0_/G_1 _arrest in HUVECs. These results were consistent with that published in our previous reports [19-21].

### DIF-1 induced proteolysis of cyclin D1 in HUVECs

**Figure 2:**
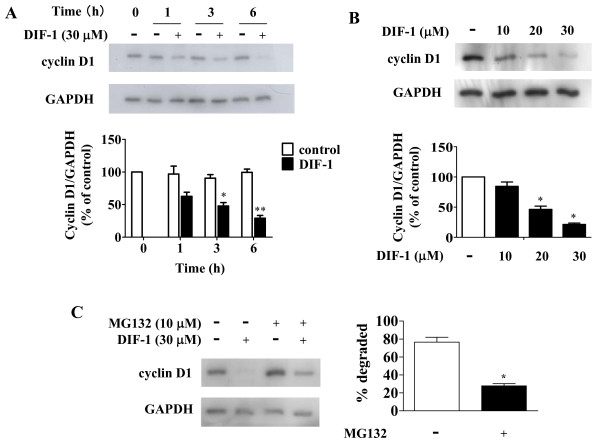
**The effect of DIF-1 on cyclin D1 protein amount**. (A) Time-course. HUVECs were incubated with or without DIF-1 (30 μM) for the periods indicated. Samples were subjected to Western blot analysis using the anti-cyclin D1 antibody. The membrane was re-probed with the anti-GAPDH antibody. The levels of protein bands were quantified and shown as percentages of the control level at time 0. Values are mean ± SE of three independent experiments. The asterisk indicates *P < 0.01 and **P < 0.001 versus control. (B) Dose dependency. HUVECs were incubated with or without various amount of DIF-1 for 4 h. Samples were subjected to Western blot analysis using the anti-cyclin D1 antibody. The membrane was re-probed with the anti-GAPDH antibody. The levels of protein bands were quantified and shown as percentages of the control level. Values are mean ± SE of three independent experiments. The asterisk indicates *P < 0.001 versus control. (C) Effect of MG132. HUVECs were pretreated with MG132 (10 μM) for 3 h and incubated with or without DIF-1 (30 μM) for 6 h. Samples were subjected to Western blot analysis using the anti-cyclin D1 antibody and the membrane was re-probed with the anti-GAPDH antibody. The levels of protein bands are quantified and shown as percentages of the degraded amounts. Values are mean ± SE of three independent experiments. The asterisk indicates *P < 0.01 and versus control.

We previously reported that DIF-1 had strong effects on cyclin D1 protein level [21,22]. Therefore, we examined the effects of DIF-1 on cyclin D1 protein quantity using HUVECs. DIF-1 rapidly reduced the protein level of cyclin D1 in time- and dose-dependent manners (Figure 2A and 2B, respectively). Next, we examined the effects of proteasome inhibitor MG132, since cyclin D1 has been reported to be degraded by ubiquitin-dependent proteolysis. MG132 significantly attenuated the effects of DIF-1, indicating that DIF-1 induced proteolysis of cyclin D1 in HUVECs (Figure 2C).

### DIF-1 inhibited angiogenesis *in vitro*

**Figure 3:**
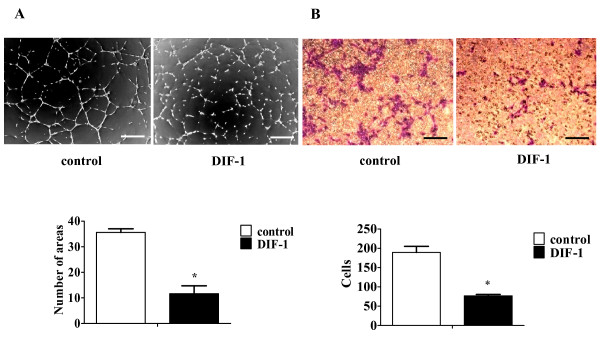
**DIF-1 inhibited *in vitro *angiogenesis**. (A) DIF-1 inhibited tube formation of HUVECs. HUVECs were seeded in Matrigel-coated well with or without DIF-1 30 μM and incubated for 8 h. The tube formation of control HUVECs was normalized as 100%. Each value represents the mean ± SE of three independent experiments. The asterisk indicates *P < 0.01 versus control. The scale bars indicate 200 μm. (B) DIF-1 inhibited the migration of HUVECs. HUVECs were seeded into the upper part of Boyden chamber. The lower compartments were filled with 600 μl of DMEM supplemented with 0.1% bovine serum albumin and 20 ng/ml (0.52 nM) VEGF. After HUVECs were incubated for 10 h, the migrated cells on the lower surface of the membrane were quantified by counting the number of cells in ten random fields per membrane and expressed as cells/fields (mean ± SE). Data are representative of three other experiments. The asterisk indicates *P < 0.01 versus control. The scale bars indicate 500 μm.

To evaluate the effects of DIF-1 on angiogenesis *in vitro*, we performed tube formation assay. HUVECs formed blood vessel-like structure (tubes) on Matrigel-coated wells following incubation for 8 h. However, DIF-1-treated HUVECs almost failed to form vessel-like structures and the number of areas surrounded by tubes was significantly smaller than that in control cells (Figure 3A). Subsequently, the effects of DIF-1 on HUVECs migration were evaluated using a Boyden Chamber. Although the cells migrated into the lower chamber even in the absence of VEGF, the number of migrating cells increased by about 30% when VEGF was added to the lower chamber. Therefore, VEGF was added to the lower chamber when the effect of DIF-1 was investigated. Although VEGF in the lower chamber induced cell migration after incubation for 10 h, DIF-1 significantly reduced the number of migrated cells (Figure 3B). These results clearly indicated that DIF-1 inhibited angiogenesis *in vitro*.

### DIF-1 inhibited angiogenesis *in vivo*

**Figure 4:**
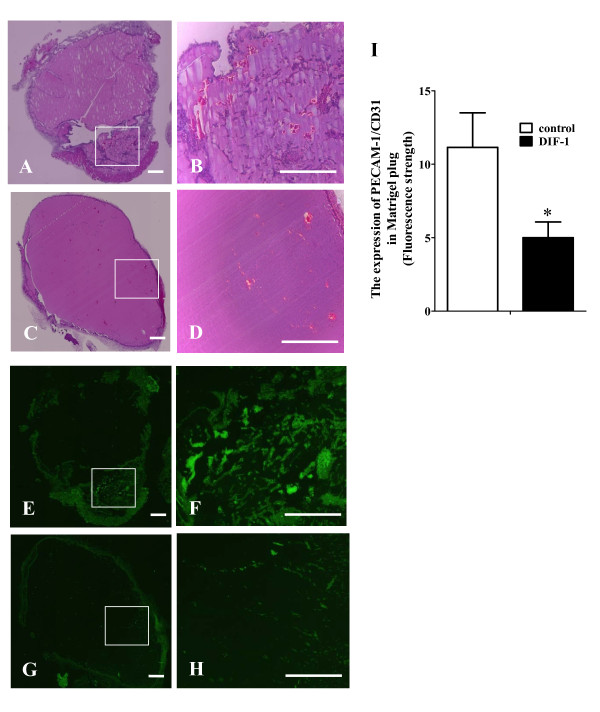
**DIF-1 suppressed VEGF-induced angiogenesis in Matrigel plug**. VEGF-containing Matrigel was injected subcutaneously into the flanks of 6-week old C57/BL6 mice. Seven days later, Matrigel plug was extracted and embedded in paraffin. Sections were stained with hematoxylin-eosin staining (A-D) and immunofluorescence staining using PECAM-1(CD31) (E-H) (Higher magnification of the boxed areas in A, C, E and G are shown in B, D, F and H, respectively). The scale bars indicate 500 μm. (I) The expression of PECAM-1 (CD31) was analyzed with fluorescence microscopy and expressed as the strength of fluorescence in Matrigel-plug (mean ± SE) of three independent experiments. The asterisk indicates *P < 0.05 versus control.

We examined the effects of DIF-1 on angiogenesis *in vivo *by Matrigel plug assay. VEGF containing-Matrigel was prepared with or without DIF-1 (30 μM) and injected into the flanks of mice. As shown in Figure 4, blood vessels indicated by the expression of PECAM-1/CD31, an endothelial cell-specific antigen, were strongly induced into the injected Matrigel plugs. On the other hand, the level of PECAM-1/CD31 expression was significantly lower in DIF-1-containing Matrigel plugs than in the control plugs, indicating the presence of fewer blood vessels.

**Figure 5:**
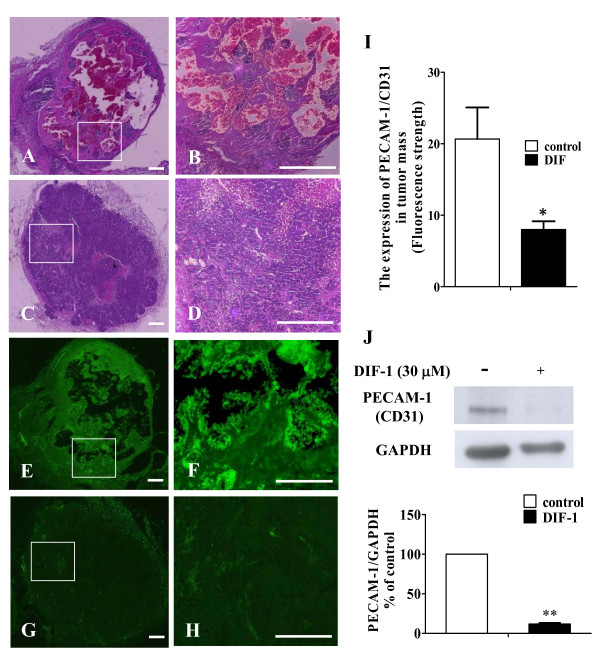
**DIF-1 inhibited angiogenesis in xenograft tumor**. (A-I) DIF-1 suppressed human cervical carcinoma cell line (HeLa)-induced angiogenesis. HeLa cells mixed with liquid Matrigel in the presence or absence of 30 μM of DIF-1 were injected subcutaneously into the flanks of nude mice. The removed tumor sections were stained with hematoxylin-eosin staining (A-D) and immunofluorescence staining using PECAM-1(CD31) (E-H) (Higher magnification of the boxed areas in A, C, E and G are shown in B, D, F and H, respectively). The scale bars indicate 500 μm. (I) The expression of PECAM-1 (CD31) was analyzed with fluorescence microscopy and expressed as the mean strength of fluorescence in Matrigel-plug (mean ± SE) of three independent experiments. (J) DIF-1 suppressed PECAM-1 protein expression in the removed tumors. The samples of the removed tumor were subjected to Western blot analysis using the anti-PECAM-1 (CD31) antibody. The membrane was re-probed with the anti-GAPDH antibody. The levels of protein bands were quantified and are shown as percentage of the control level. Values are mean ± SE of three independent experiments. The asterisk indicates *P < 0.05, **P < 0.001 versus control.

Subsequently, we examined the effects of DIF-1 on tumor-induced angiogenesis using HeLa cells. Two weeks after injection, Matirgel/HeLa cell mixture formed tumor mass and blood vessels were grown into the mass. Immunohistochemical analysis of PECAM-1/CD31 in tumor masses clearly showed that the content of blood vessels was much less in DIF-1-containing masses (Figure 5A-I). This finding was also confirmed by Western blot analysis. As shown in Figure 5J, the level of PECAM-1/CD31 expression was significantly lower in DIF-1-containing masses than in the control masses. These results indicated that DIF-1 inhibited blood vessel growth induced by VEGF and tumor cells.

### DIF-1 decreased VEGFR-1 and VEGFR-2 protein expression

**Figure 6:**
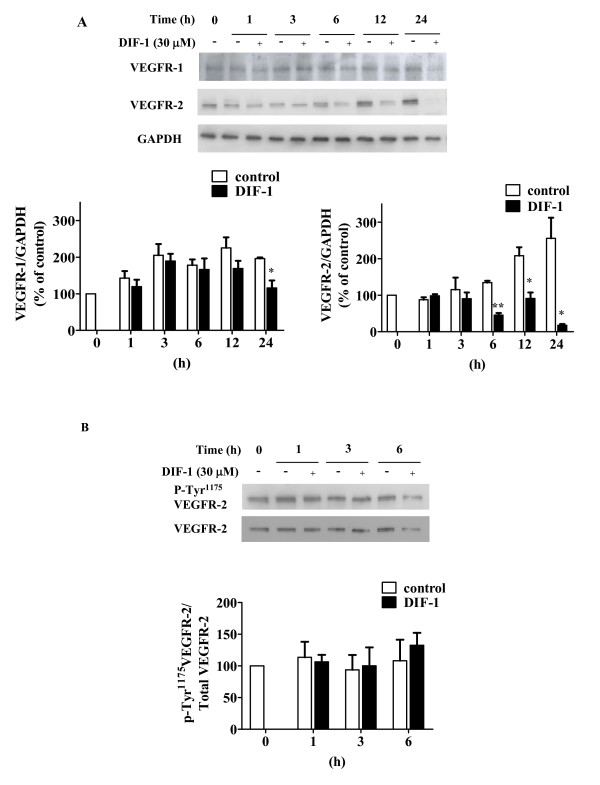
**The effects of DIF-1 on VEGF receptors, VEGFR-1 and VEGFR-2**. (A) DIF-1 decreased VEGFR-1 and VEGFR-2 expression in HUVECs. HUVECs were incubated with or without DIF-1 (30 μM) for the periods indicated and the samples were subjected to Western blot analysis for VEGFR-1 and VEGFR-2. The levels of protein bands were quantified and are shown as percentage of the control level at time 0. Values are mean ± SE of three independent experiments. The asterisk indicates *P < 0.05 **P < 0.001 versus control. (B) DIF-1 did not affect phosphorylation level of VEGFR-2. HUVECs were incubated with or without DIF-1 (30 μM) for the periods indicated and the samples were subjected to Western blot analysis using anti-phospho-VEGFR-2 (Tyr^1175^) antibody. The membrane was re-probed with anti-VEGFR-2 antibody. The levels of protein bands were quantified and are shown as percentage of the control level at time 0. Values are mean ± SE of three independent experiments.

It is well known that VEGF-A, a major regulator for angiogenesis, binds to VEGFR-1 (Flt-1) and VEGFR-2 (KDR/Flk-1) to transduce its signal. To clarify the mechanism by which DIF-1 suppresses angiogenesis, first we examined the effects of DIF-1 on VEGFR-1 and VEGFR-2 expression in HUVECs by Western blot analysis. As shown in Figure 6A, although DIF-1 significantly reduced the expression of both receptors in HUVECs, the effect was much stronger in VEGFR-2 than VEGFR-1.

VEGF increases VEGFR-2 phosphorylation on Tyr^1175 ^for activation [29-31]. Although DIF-1 decreased the phosphorylation level of Tyr^1175 ^on VEGFR-2 in a time-dependent manner, it was parallel with the time course of the VEGFR-2 protein amount, indicating that DIF-1 had no significant effects on the level of VEGFR-2 phosphorylation (Figure 6B).

### DIF-1 reduced VEGFR-2 protein synthesis

**Figure 7:**
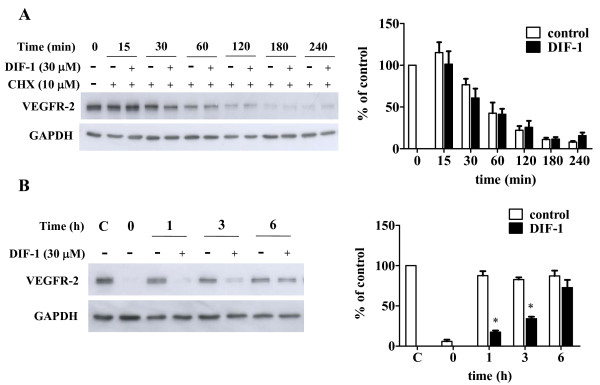
**The effects of DIF-1 on the proteolysis and the proteosynthesis of VEGFR-2**. (A) DIF-1 did not affect the degradation speed of VEGFR-2. HUVECs were incubated with or without DIF-1 (30 μM) for the given periods in the presence of cycloheximide (10 μM). Protein samples were subjected to Western blot analysis for VEGFR-2. The membrane was re-probed with anti-GAPDH antibody. The levels of protein bands were quantified and are shown as percentage of the control level at time 0. Values are mean ± SE of three independent experiments. (B) DIF-1 attenuated the protein synthesis of VEGFR-2. After HUVECs were pretreated with cycloheximide (10 μM) for 3 h, the medium was changed to fresh medium and cells were incubated with or without DIF-1 (30 μM) for the given periods. Protein samples were subjected to Western blot analysis for VEGFR-2. The membrane was re-probed with anti-GAPDH antibody. The levels of protein bands were quantified and are shown as percentage of the control cells which were not treated with cycloheximide (lane 1). Values are mean ± SE of three independent experiments. The asterisk indicates *P < 0.001 versus control.

To clarify the mechanism of DIF-1-induced VEGFR-2 protein suppression, we first examined the effects of DIF-1 on VEGFR-2 degradation using protein synthesis inhibitor cycloheximide. As shown in Figure 7A, DIF-1 did not accelerate reduction in VEGFR-2 protein quantity, indicating that DIF-1 had no significant effects on VEGFR-2 proteolysis.

Next, we investigated VEGFR-2 protein synthesis. HUVECs were pretreated with cycloheximide for 3 h and then the medium was changed to fresh growth medium to wash out cycloheximide. As shown in Figure 7B, after VEGFR-2 protein disappeared following cycloheximide treatment, it was rapidly restored and reached a plateau after 1 h incubation. However, restoration of VEGFR-2 protein was significantly delayed by DIF-1 treatment.

### DIF-1 suppressed VEGFR-2 mRNA level and VEGFR-2 promoter activity

**Figure 8:**
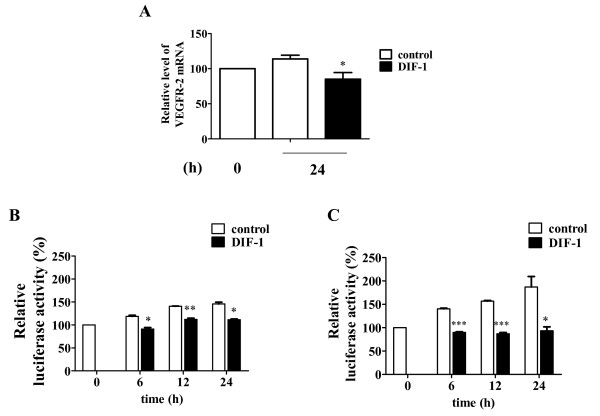
**DIF-1 reduced VEGFR-2 mRNA level and promoter activity**. (A) The effects of DIF-1 on the VEGFR-2 mRNA levels. Total RNAs were extracted from HUVECs treated with or without DIF-1 (30 μM) for 24 h. The VEGFR-2 mRNA levels were determined by TaqMan quantitative real-time RT-PCR. Values are mean ± SE of four independent experiments. The asterisk indicates *P < 0.05 versus control. (B and C) The effects of DIF-1 on the human VEGFR-2 gene promoter activity on HUVECs (B) or BAECs (C). HUVECs or BAECs were co-transfected with VEGFR-2 pGL-3 and pRL-SV40. After 24 h incubation, HUVECs and BAECs were stimulated with or without DIF-1 (30 μM) for the given periods. Luciferase activity is shown as percentages of the control level at time 0. Values are mean ± SE of three independent experiments. The asterisk indicates *P < 0.01 **P < 0.001 ***P < 0.0001 versus control.

Subsequently, we examined the effect of DIF-1 on the mRNA expression of VEGFR-2 in HUVECs by real-time PCR analysis and found that DIF-1 significantly suppressed VEGFR-2 mRNA level (Figure 8A). We further examined the effects of DIF-1 on human VEGFR-2 gene promoter activity using a luciferase reporter plasmid. Since the efficiency of DNA transfection in HUVECs was low (10% to 20%), we also employed BAECs in which transfection efficiency was much higher (60% to 70%) [32]. As shown in Figure 8B and 8C, luciferase reporter activity driven by the 5'-flanking region of human VEGFR-2 gene in HUVECs (B) or BAECs (C) was increased as incubation proceeded. However, the promoter activity was not significantly increased in DIF-1-treated HUVECs and BAECs. Therefore DIF-1 appeared to suppress VEGFR-2 protein and mRNA expressions by inhibiting the promoter activity in HUVECs.

### Wnt-3a suppressed VEGFR-2 promoter activity

**Figure 9:**
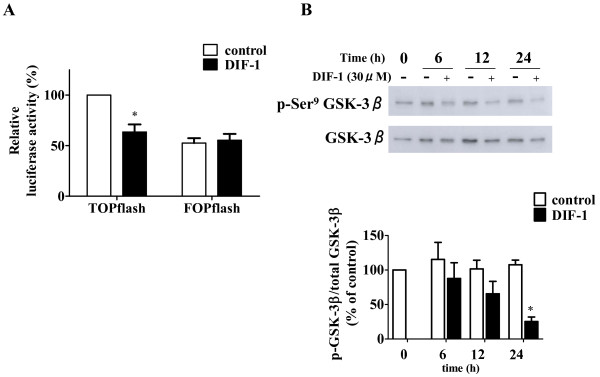
**DIF-1 suppressed Wnt/β-catenin signaling pathway in HUVECs**. (A) Effect of DIF-1 on TCF transcriptional activity. TOPflash or FOPflash was co-transfected with pRL-SV40 into HUVECs. After 24 h incubation, HUVECs were stimulated with or without DIF-1 (30 μM) for 24 h. Luciferase activity is shown as percentages of the control level. Values are mean ± SE of three independent experiments. The asterisk indicates *P < 0.01 versus control. (B) DIF-1 activated GSK-3β. HUVECs were incubated with or without DIF-1 (30 μM) for given periods. Protein samples were subjected to Western blot analysis for phosphorylation level of Ser^9 ^on GSK-3β. The membrane was reprobed with the anti-GSK-3β antibody. The levels of protein bands were quantified and are shown as percentage of the control level at time 0. Values are mean ± SE of three independent experiments. The asterisk indicates *P < 0.0001 versus control.

As previously reported, DIF-1 suppressed the Wnt/β-catenin signaling pathway by activating GSK-3β in tumor cells. Moreover, the Wnt/β-catenin signaling pathway has been shown to play an important role in promoting angiogenesis. Therefore, we examined the involvement of the Wnt/β-catenin signaling pathway in DIF-1-induced VEGFR-2 suppression. As shown in Figure 9A, DIF-1 significantly inhibited TOPflash (TCF reporter plasmid) activity, whereas it did not affect FOPflash (negative control) activity. Furthermore, as shown in Figure 9B, DIF-1 reduced the phosphorylation level of Ser^9 ^on GSK-3β, indicating that DIF-1 certainly inhibited the Wnt/β-catenin signaling pathway by activating GSK-3β in HUVECs.

**Figure 10:**
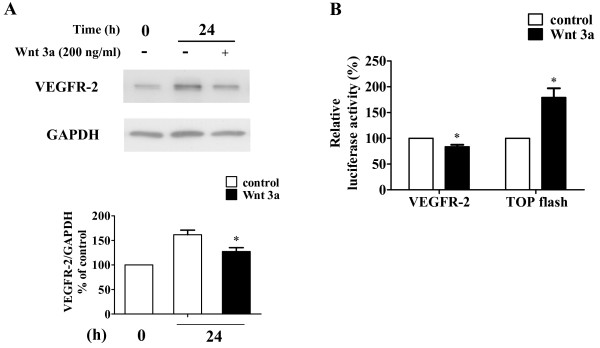
**Effects of Wnt3a on VEGFR-2**. (A) Wnt3a suppressed VEGFR-2 expression. HUVECs were incubated with or without Wnt3a (200 ng/ml = 5.35 nM) for 24 h and the samples were subjected to Western blot analysis for VEGFR-2. The levels of protein bands were quantified and are shown as percentage of the control level at time 0. Values are mean ± SE of three independent experiments. The asterisk indicates *P < 0.05 versus control. (B) Wnt3a suppressed the VEGFR-2 promoter activity. HUVECs were co-transfected with VEGFR-2 pGL-3 or TOPflash and pRL-SV40. After 24 h incubation, cells were stimulated with or without Wnt3a (200 ng/ml) for 12 h. Luciferase activity is shown as percentages of the control. Values are mean ± SE of three independent experiments. The asterisk indicates *P < 0.05 versus control.

Although VEGF is one of the target genes of the Wnt/β-catenin signaling pathway [33,34], it has not been elucidated whether VEGFR-2 gene also belongs to the target genes of this signaling pathway. Therefore, the effects of Wnt3a as an activator of the Wnt/β-catenin signaling pathway on VEGFR-2 protein expression were examined. Although the amount of VEGFR-2 protein was increased after a 24 h-incubation period, treatment with Wnt3a suppressed VEGFR-2 protein increase (Figure 10A). This observation was confirmed by the luciferase reporter assay using the 5'-flanking region of the human VEGFR-2 gene. As shown in Figure 10B, Wnt3a suppressed VEGFR-2 promoter activity slightly but significantly in HUVECs, whereas it clearly increased TOPflash activity. These results indicated that Wnt3a suppressed VEGFR-2 expression via inhibition of promoter activity, suggesting that VEGFR-2 gene expression was suppressed by activating the Wnt/β-catenin signaling pathway.

Taken together, although DIF-1 suppressed the Wnt/β-catenin signaling pathway in HUVECs as well as tumor cells, inhibition of VEGFR-2 promoter activity induced by DIF-1 was not due to suppression of this signaling pathway.

## Discussion

In this study, we demonstrated that DIF-1 strongly inhibited angiogenesis *in vitro *and *in vivo*. As it is known that VEGF-A signal plays a prominent role in angiogenesis, we paid special attention to two types of VEGF receptors, VEGFR-1 and VEGFR-2. Although DIF-1 decreased the levels of protein expression of both receptors on HUVECs, the effects were faster and stronger in VEGFR-2 than VEGFR-1. Activation of VEGFR-2 by VEGF-A depends on the phosphorylation status of several tyrosine residues (such as 951, 1059, 1175, and 1214) in VEGFR-2. Among these tyrosine residues, Tyr^1175 ^is the binding site of phospholipase-Cγ, a main signal transducer of VEGFR-2 [32-34]. However, as shown in Figure 6B, DIF-1 did not have significant effects on the Tyr^1175 ^phosphorylation status, suggesting that DIF-1 did not affect VEGFR-2 activation. Since VEGFR-2 is a direct signal transducer for pathological angiogenesis as observed in cancers, the powerful reduction of VEGFR-2 protein levels may be involved in DIF-1 induced anti-angiogenic effects.

We also attempted to clarify the mechanism by which DIF-1 reduced the amount of VEGFR-2 protein. DIF-1 affected the synthesis rather than proteolysis of VEGFR-2. This was consistent with the result that DIF-1 inhibited the mRNA expression and promoter activity of VEGFR-2. However, degrees of suppression of the mRNA expression (29%) and promoter activity (24%) were relatively small compared to VEGFR-2 protein quantity suppression (93%) after 24 h-treatment with DIF-1. The same sort of phenomenon was also observed by Wnt3a (16% promoter activity suppression vs. 34% protein quantity suppression). Although we could not explain this difference at present, the short half-life of VEGFR-2 protein of about 1 h [35] could be associated with this phenomenon. In other words, as the proteolysis of VEGFR-2 is quick and rapid synthesis is required to restore VEGFR-2, even weak inhibition of promoter activity may significantly affect the quantity of VEGFR-2 protein.

Since we have shown that DIF-1 inhibits the Wnt/β-catenin signaling pathway in various cells, the effects of DIF-1 on the Wnt/β-catenin signaling pathway in HUVECs were examined. We found that DIF-1 also inhibited this signaling pathway via GSK-3β activation in HUVECs. Although the Wnt/β-catenin signaling pathway has been reported to be important to promote angiogenesis *in vitro *[24-28], the role of Wnt/β-catenin signaling pathway in endothelial cells and angiogenesis is controversial. Cheng *et al*. reported that Wnt1 signaling inhibits HUVEC proliferation [36]. On the other hand, it has been reported that Wnt1 and 3a mediated induction of VEGFR-2 (Quek-1) expression during avian somite development [37]. In this study, we showed that Wnt3a slightly but significantly reduced promoter activity and VEGFR-2 protein expression. Therefore, suppression of VEGFR-2 expression induced by DIF-1 may not be due to suppression of the Wnt/β-catenin signaling pathway. Our results might suggest that activation of the Wnt/β-catenin signaling pathway suppressed the promotion of angiogenesis. However, Samarzija *et al*. showed that although Wnt3a stimulated HUVEC proliferation and migration independent of VEGFR signaling [38]. Therefore, further studies are needed to elucidate the relationship between the Wnt/β-catenin signaling pathway and angiogenesis.

Cyclin D1 plays a key role in the initiation and progression of the G_1 _phase [39]. We previously showed that DIF-1 and DIF-3 reduced cyclin D1 quantity and induced cell cycle arrest in G_0_/G_1 _phase using various mammalian cells [19,21,22]. In this study, we also demonstrated that DIF-1 inhibited HUVECs proliferation and induced restriction of cell cycle in the G_0_/G_1 _phase by degrading cyclin D1. This result is consistent with that published in our previous reports, and indicates that cyclin D1 also plays an important role in HUVEC proliferation. Furthermore, it has been reported that antisense to cyclin D1 inhibited tumor-associated neovascularization [40]. As such, suppression of cyclin D1 expression may be one of the anti-angiogenesis mechanisms induced by DIF-1.

## Conclusions

In summary, we found that DIF-1 reduced the expression of cyclin D1 and VEGFR-2 in HUVECs. The reduction of cyclin D1 and VEGFR-2 expression may inhibit proliferation, and reduction of VEGFR-2 may cause inhibition of migration and tube formation. These effects may explain the powerful anti-angiogenic properties of DIF-1.

We previously reported that DIF-1 showed anti-tumor activity by inhibiting cyclin D1 expression and the Wnt/β-catenin signaling pathway. In addition to these effects, this study demonstrated that DIF-1 also exhibited anti-angiogenic effects independent of the Wnt/β-catenin signaling pathway. Elucidation of the target molecule of DIF-1 will facilitate the development of potent novel anti-tumor agents which suppresses not only the Wnt/β-catenin signaling pathway but also angiogenesis.

## Methods

### Cell Culture

Human umbilical vein endothelial cells (HUVECs) were purchased from DS Pharma Biomedical (Osaka, Japan). The cells were grown in Dulbecco's modified Eagle's medium (Sigma-Aldrich, St Louis, Mo, USA) supplemented with 20% fetal bovine serum, 5 ng/ml (0.29 nM) recombinant human basic fibroblast growth factor (PeproTech, Rocky Hill, NJ, USA), 100 units/ml penicillin G, and 100 μg/ml streptomycin using 0.1% gelatin coated dishes. HeLa cells (human cervical carcinoma cell line) and bovine aortic endothelial cells (BAECs) were grown in Dulbecco's modified Eagle's medium (DMEM) (Sigma-Aldrich) supplemented with 10% fetal bovine serum, 100 units/ml penicillin G and 100 μg/ml streptomycin.

### Reagents and antibodies

DIF-1 (1-(3,5-dichloro-2, 6-dihydroxy-4-methoxyphenyl)-1-hexanone) was synthesized as described previously [41]. MG132 was obtained from the Peptide Institute (Osaka, Japan). Cycloheximide was obtained from Sigma-Aldrich. Polyclonal anti-cyclin D1 antibody, polyclonal anti-PECAM-1 (CD31) antibody and the polyclonal anti-VEGFR-1/Flt-1 antibody were purchased from Santa Cruz Biotechnology (CA, USA). Monoclonal anti-VEGFR-2 antibody and the monoclonal anti-phospho-VEGFR-2 (Tyr^1175^) antibody were from Cell Signaling Technology (Danvers, MA, USA). The monoclonal GAPDH antibody was obtained from Abcam (Cambridge, MA, USA). Growth factor reduced Matrigel was obtained from BD Biosciences (San Jose, CA, USA). TOPflash (TCF reporter plasmid) and FOPflash (negative control of TOPflash) were purchased from Upstate Biotechnology (Lake Placid, NY, USA). Human Wnt3a was from R&D Systems (Minneapolis, MN, USA).

### Cell proliferation assay

The cells were plated on 24-well plates (1.0×10^4 ^cells/well) and treated with or without various amounts of DIF-1 for defined periods. Cells were harvested by trypsin/EDTA treatment and enumerated using Coulter Counter (Beckman Coulter, Brea, CA, USA).

### Flow Cytometry

Cells harvested by trypsin/EDTA treatment were suspended in hypotonic fluorochrome solution containing 50 μg/ml of propidium iodide, 0.1% sodium citrate, and 0.1% Triton X-100. Cells (5×10^3^) for each sample were analyzed for fluorescence by a Becton-Dickinson FACScalibur (Franklin Lakes, NJ, USA).

### Western blotting

Samples were separated by 10 or 12% SDS-PAGE and transferred to a polyvinylidene difluoride membrane using a semidry transfer system (1 h, 12 V). After blocking with 5% skim milk, the membrane was probed with a first antibody. Incubation was carried out overnight at 4°C. The membrane was then washed three times and incubated with horseradish peroxidase-conjugated anti-rabbit IgG or anti-mouse IgG (Bio-Rad, Hercules, CA, USA) for 1 h. Immunoreactive proteins on the membrane were visualized by treatment with a detection reagent (LumiGLO, Cell Signaling Technology). Optical densitometric scan was performed using NIH Image J software.

### Tube formation assay

Tube formation assay was performed as previously described [42] with slight modification. Briefly, Matrigel was thawed at 4°C and 250 μl of the solution were added to each well in a 24-well plate and formed a gel at 37°C for 30 min. HUVECs were suspended at 3×10^4 ^cells in 500 μl of 3% FBS with or without 30 μM DIF-1, and then added to each well. After 8 h-incubation, the degree of tube formation was determined by counting the number of areas surrounded by tubes contained in 10 random fields, and expressed as mean ± SE.

### Cell migration assay

The effect of DIF-1 treatment on *in vitro *migration of HUVECs was determined using a Boyden Chamber [43]. The PET membrane (8 μm pore size, Greiner Bio-One, Frickenhausen, Germany) was pre-coated with 10 μg of Matrigel. HUVECs were suspended at 5×10^4 ^cells in 100 μl of serum free DMEM with or without 30 μM DIF-1 and seeded into the upper part of each chamber, whereas the lower compartments were filled with 600 μl of DMEM supplemented with 0.1% bovine serum albumin and 20 ng/ml (0.52 nM) VEGF. After incubation for 10 h at 37°C, non-migrated cells were scraped off with a cotton swab. Migrated cells on the lower surface of the membrane were fixed with 1% glutaraldehyde for 10 min and stained with 4% crystal violet for 30 min. HUVEC migration was quantified by counting the number of cells in ten random fields per membrane. Data are expressed as mean ± SE of cells/fields.

### *In vivo *mouse Matrigel-plug assay

*In vivo *angiogenesis was assayed as growth of blood vessels from mouse subcutaneous tissue into the exogenous Matrigel plug induced by VEGF or tumor cells [43]. For the analysis of VEGF-induced angiogenesis, Matrigel was prepared with 100 ng/ml (2.62 nM) VEGF, 20 units/ml heparin in the presence or absence of 30 μM DIF-1 at 4°C. The liquid Matrigel was injected (final volume; 500 μl) into the flanks of C57BL/6 mice (5~7 weeks, n = 7 for each group) using a cold syringe and allowed to polymerize into a solid gel by body temperature. Seven days later, Matrigel plugs were extracted and samples were prepared for immunohistochemical analysis. To analysis for tumor-induced angiogenesis, 1×10^6 ^HeLa cells were mixed with liquid Matrigel in the presence or absence of 30 μM DIF-1 (final volume; 500 μl). The mixture was injected subcutaneously in the flanks of 6 week-old nude mice (Kyudo, Saga, Japan). Two weeks later, the tumors were removed and samples were prepared for Western blot and immunohistochemical analyses. The handling and sacrificing of all animals were carried out in accordance with nationally prescribed guidelines, and ethical approval for studies was granted by the Animal Care and Use Committee of Kyushu University.

### Immunohistochemical analysis

The removed Matrigel plugs and tumors were fixed in 10% buffered formalin followed by embedding in paraffin. Sections were then stained with hematoxylin-eosin staining and immunofluorescence staining. For immunofluorescence staining, primary PECAM-1/CD31 antibody (1:50 dilution) was applied to the sections and the slides were incubated overnight at 4°C. The secondary antibody (Histofine, Nichirei, Tokyo, Japan) was applied to the sections and incubated for 1 h. The slides were subsequently incubated with streptavidin-FITC (Invitrogen, Carlsbad, CA, USA) and the fluorescence strength was analyzed with Biozero fluorescence microscopy (Keyence, Osaka, Japan).

### Real-time quantitative reverse transcriptase-polymerase chain reaction

Total RNAs were extracted from HUVECs using TRIzol (Invitrogen) and SV total RNA isolation system (Promega, Madison, WI, USA). First-strand cDNAs were synthesized from 2 μg of total RNA using a high-capacity cDNA reverse transcription kit (Applied Biosystems, Carlsbad, CA, USA). The 100 ng cDNA products were used for quantitative real-time PCR performed using TaqMan Universal PCR Master Mix (Applied Biosystems) and TaqMan MGB primers [VEGFR-2 (Hs00911700_m1) and GAPDH (Hs99999905_m1)] with an ABI Prism 7500 (Applied Biosystems). The following PCR conditions were used: 50°C for 2 minutes, then 95°C for 10 minutes, followed by 40 cycles at 95°C for 15 seconds and 60°C for 1 minute. Cycle threshold (C_T_) values for each gene were obtained for each sample. Differences in C_T _values between VEGFR-2 gene and endogenous control (GAPDH) were calculated and used for statistical analyses.

### Construction of reporter plasmid

The 5'-flanking region of human VEGFR-2 [44] was amplified and cloned into PCR 2.1 (Invitrogen) for DNA sequencing. After confirming the sequence, DNA fragments (-1003/-48 bp relative to the transcription start site) were excised with *Sac*I and *Bgl*II and cloned into pGL3-Basic vectors (Promega).

### Luciferase reporter gene assay

Cells were transiently transfected with plasmid DNA (TOPflash, FOPflash orVEGFR-2/pGL-3) and pRL-SV40, a Renilla luciferase expression plasmid (Promega) to control transfection efficacy, using Superfect reagent (Qiagen, Hilden, Germany). To measure luciferase activities, Dual-luciferase Reporter Assay (Promega) and a luminometer (Lumat LB 9507; Berthold Technologies, Bad Wildbad, Germany) were used. Firefly luciferase activities were normalized to that of Renilla luciferase.

### Statistics

The results are expressed as mean ± SE. Statistical analysis of the differences between values were conducted using the Student's *t*-test or the one-way ANOVA with Bonferroni post-hoc tests (GraphPad Prism 5.0, GraphPad Software, La Jolla, CA, USA). A *P *value < 0.05 was considered statistically significant.

## Abbreviations

HUVEC: human umbilical vein endothelial cell; BAEC: bovine aortic endothelial cell; VEGF: vascular endothelial growth factor; VEGFR: vascular endothelial growth factor receptor; DIF: differentiation-inducing factor; GSK-3β: glycogen synthase kinase-3β

## Competing interests

The authors declare that they have no competing interests.

## Authors' contributions

TY contributed to the major part of experimental work, analyzed and interpreted data, performed the statistics and drafted the manuscript. FT conceived the study, participated in its design and data analysis, and contributed with scientific discussion and manuscript preparation. FS contributed the production of reporter plasmid. YW provided DIF-1. SM, MH and SH interpreted data and contributed with scientific discussion. TS supervised the project and helped draft the manuscript. All authors read and approved the final manuscript.

## References

[B1] Conway EM Collen D Carmeliet P Molecular mechanisms of blood vessel growth Cardiovasc Res 2001 49 507 521 10.1016/S0008-6363(00)00281-9 11166264

[B2] Chavakis E Dimmeler S Regulation of endothelial cell survival and apoptosis during angiogenesis Arterioscler Thromb Vasc Biol 2002 22 887 893 10.1161/01.ATV.0000017728.55907.A9 12067894

[B3] Gupta MK Qin RY Mechanism and its regulation of tumor-induced angiogenesis World J Gastroenterol 2003 9 1144 1155 12800214 10.3748/wjg.v9.i6.1144 PMC4611774

[B4] Wong ML Prawira A Kaye AH Hovens CM Tumour angiogenesis: its mechanism and therapeutic implications in malignant gliomas J Clin Neurosci 2009 16 1119 1130 10.1016/j.jocn.2009.02.009 19556134

[B5] Ferrara N Molecular and biological properties of vascular endothelial growth factor J Mol Med 1999 77 527 543 10.1007/s001099900019 10494799

[B6] Zachary I VEGF signalling: integration and multi-tasking in endothelial cell biology Biochem Soc Trans 2003 31 1171 1177 10.1042/BST0311171 14641020

[B7] Olsson AK Dimberg A Kreuger J Claesson-Welsh L VEGF receptor signalling-in control of vascular function Nat Rev Mol Cell Biol 2006 7 359 371 10.1038/nrm1911 16633338

[B8] Hicklin DJ Ellis LM Role of the vascular endothelial growth factor pathway in tumor growth and angiogenesis J Clin Oncol 2005 23 1011 1027 10.1200/JCO.2005.06.081 15585754

[B9] De Vries C Escobedo JA Ueno H Houck K Ferrara N Williams LT The fms-like tyrosine kinase, a receptor for vascular endothelial growth factor Science 1992 255 989 991 10.1126/science.1312256 1312256

[B10] Quinn TP Peters KG De Vries C Ferrara N Williams LT Fetal liver kinase 1 is a receptor for vascular endothelial growth factor and is selectively expressed in vascular endothelium Proc Natl Acad Sci USA 1993 90 7533 7537 10.1073/pnas.90.16.7533 8356051 PMC47176

[B11] Roskoski R Jr VEGF receptor protein-tyrosine kinases: Structure and regulation Biochem Biophys Res Commun 2008 375 287 291 10.1016/j.bbrc.2008.07.121 18680722

[B12] Rahimi N Dayanir V Lashkari K Receptor chimeras indicate that the vascular endothelial growth factor receptor-1 (VEGFR-1) modulates mitogenic activity of VEGFR-2 in endothelial cells J Biol Chem 2000 275 16986 16992 10.1074/jbc.M000528200 10747927

[B13] Shibuya M Differential roles of vascular endothelial growth factor receptor-1 and receptor-2 in angiogenesis J Biochem Mol Biol 2006 39 469 478 17002866 10.5483/bmbrep.2006.39.5.469

[B14] Kowanetz M Ferrara N Vascular endothelial growth factor signaling pathways: therapeutic perspective Clin Cancer Res 2006 12 5018 5022 10.1158/1078-0432.CCR-06-1520 16951216

[B15] Petrova TV Makinen T Alitalo K Signaling via vascular endothelial growth factor receptors Exp Cell Res 1999 253 117 130 10.1006/excr.1999.4707 10579917

[B16] Shibuya M Claesson-Welsh L Signal transduction by VEGF receptors in regulation of angiogenesis and lymphangiogenesis Exp Cell Res 2006 312 549 560 10.1016/j.yexcr.2005.11.012 16336962

[B17] Morris HR Taylor GW Masento MS Jermyn KA Kay RR Chemical structure of the morphogen differentiation inducing factor from Dictyostelium discoideum Nature 1987 328 811 814 10.1038/328811a0 3627228

[B18] Kubohara Y DIF-1, putative morphogen of D. discoideum, suppresses cell growth and promotes retinoic acid-induced cell differentiation in HL-60 Biochem Biophys Res Commun 1997 236 418 422 10.1006/bbrc.1997.6964 9240452

[B19] Miwa Y Sasaguri T Kosaka C Taba Y Ishida A Abumiya T Kubohara Y Differentiation-inducing factor-1, a morphogen of dictyostelium, induces G_1 _arrest and differentiation of vascular smooth muscle cells Circ Res 2000 86 68 75 10625307 10.1161/01.res.86.1.68

[B20] Takahashi-Yanaga F Taba Y Miwa Y Kubohara Y Watanabe Y Hirata M Morimoto S Sasaguri T Dictyostelium differentiation-inducing factor-3 activates glycogen synthase kinase-3β and degrades cyclin D1 in mammalian cells J Biol Chem 2003 278 9663 9670 10.1074/jbc.M205768200 12522140

[B21] Mori J Takahashi-Yanaga F Miwa Y Watanabe Y Hirata M Morimoto S Shirasuna K Sasaguri T Differentiation-inducing factor-1 induces cyclin D1 degradation through the phosphorylation of Thr286 in squamous cell carcinoma Exp Cell Res 2005 310 426 433 10.1016/j.yexcr.2005.07.024 16153639

[B22] Yasmin T Takahashi-Yanaga F Mori J Miwa Y Hirata M Watanabe Y Morimoto S Sasaguri T Differentiation-inducing factor-1 suppresses gene expression of cyclin D1 in tumor cells Biochem Biophys Res Commum 2005 338 903 909 10.1016/j.bbrc.2005.10.018 16243295

[B23] Matsuzaki E Takahashi-Yanaga F Miwa Y Hirata M Watanabe Y Sato N Morimoto S Hirofuji T Maeda K Sasaguri T Differentiation-inducing factor-1 alters canonical Wnt signaling and suppressed alkaline phosphatase expression in osteoblast-like cell lines J Bone Miner Res 2006 21 1307 1316 10.1359/jbmr.060512 16869729

[B24] Goodwin AM D'Amore PA Wnt signaling in the vasculature Angiogenesis 2002 5 1 9 10.1023/A:1021563510866 12549854

[B25] Masckauchan TN Shawber CJ Funahashi Y Li CM Kitajewski J Wnt/beta-catenin signaling induces proliferation, survival and interleukin-8 in human endothelial cells Angiogenesis 2005 8 43 51 10.1007/s10456-005-5612-9 16132617

[B26] Parmalee NL Kitajewski J Wnt signaling in angiogenesis Curr Drug Targets 2008 9 558 564 10.2174/138945008784911822 18673241 PMC4052372

[B27] Zerlin M Julius MA Kitajewski J Wnt/Frizzled signaling in angiogenesis Angiogenesis 2008 11 63 69 10.1007/s10456-008-9095-3 18253847

[B28] Wang Y Nakayama N Wnt and BMP signaling are both required for hematopoietic cell development from human ES cells Stem Cell Res 2009 3 113 125 10.1016/j.scr.2009.06.001 19595658

[B29] Takahashi T Yamaguchi S Chida K Shibuya M A single autophosphorylation site on KDR/Flk-1 is essential for VEGF-A-dependent activation of PLC-γ and DNA synthesis in vascular endothelial cells EMBO J 2001 20 2768 2778 10.1093/emboj/20.11.2768 11387210 PMC125481

[B30] Rahimi N VEGFR-1 and VEGFR-2: two non-identical twins with a unique physiognomy Front Biosci 2006 11 818 829 10.2741/1839 16146773 PMC1360224

[B31] Blanes MG Oubaha M Rautureau Y Gratton JP Phosphorylation of tyrosine 801 of vascular endothelial growth factor receptor-2 is necessary for Akt-dependent endothelial nitric-oxide synthase activation and nitric oxide release from endothelial cells J Biol Chem 2007 282 10660 10669 10.1074/jbc.M609048200 17303569

[B32] Miyagi M Miwa Y Takahashi-Yanaga F Morimoto S Sasaguri T Activator protein-1 mediates shear stress-induced prostaglandin D synthase gene expression in vascular endothelial cells Arterioscler Thromb Vasc Biol 2005 25 970 975 10.1161/01.ATV.0000159702.68591.0d 15718494

[B33] Zhang X Gaspard JP Chung DC Regulation of vascular endothelial growth factor by the Wnt and K-ras pathways in colonic neoplasia Cancer Res 2001 61 6050 6054 11507052

[B34] Easwaran V Lee SH Inge L Guo L Goldbeck C Garrett E Wiesmann M Garcia PD Fuller JH Chan V Randazzo F Gundel R Warren RS Escobedo J Aukerman SL Taylor RN Fantl WJ β-Catenin regulates vascular endothelial growth factor expression in colon cancer Cancer Res 2003 63 3145 3153 12810642

[B35] Meissner M Reichenbach G Stein M Hrgovic I Kaufmann R Gille J Down-regulation of vascular endothelial growth factor receptor 2 is a major molecular determinant of proteasome inhibitor-mediated antiangiogenic action in endothelial cells Cancer Res 2009 69 1976 1984 10.1158/0008-5472.CAN-08-3150 19223539

[B36] Cheng CW Smith SK Charnock-Jones DS Wnt-1 signaling inhibits human umbilical vein endothelial cell proliferation and alters cell morphology Exp Cell Res 2003 291 415 425 10.1016/j.yexcr.2003.07.006 14644163

[B37] Nimmagadda S Geetha-Loganathan P Scaal M Christ B Huang R FGFs, Wnts and BMPs mediate induction of VEGFR-2 (*Quek-1*) expression during avian somite development Dev Biol 2007 305 421 429 10.1016/j.ydbio.2007.02.031 17425953

[B38] Samarzija I Sini P Schlange T MacDonald G Hynes NE Wnt3a regulates proliferation and migration of HUVEC via canonical and non-canonical Wnt signaling pathways Biochem Biophys Res Commun 2009 386 449 454 10.1016/j.bbrc.2009.06.033 19523451

[B39] Weinberg RA The retinoblastoma protein and cell cycle control Cell 1995 81 323 330 10.1016/0092-8674(95)90385-2 7736585

[B40] Yasui M Yamamoto H Ngan CY Damdinsuren B Sugita Y Fukunaga H Gu J Maeda M Takemasa I Ikeda M Fujio Y Sekimoto M Matsuura N Weinstein IB Monden M Antisense to cyclin D1 inhibits vascular endothelial growth factor-stimulated growth of vascular endothelial cells: implication of tumor vascularization Clin Cancer Res 2006 12 4720 4729 10.1158/1078-0432.CCR-05-1213 16899623

[B41] Masento MS Morris HR Taylor GW Johnson SJ Skapski AC Kay RR Differentiation-inducing factor from the slime mould Dictyostelium discoideum and its analogues. Synthesis, structure and biological activity Biochem J 1988 256 23 28 3223901 10.1042/bj2560023 PMC1135362

[B42] Kawasaki J Hirano K Hirano M Nishimura J Nakatsuka A Fujishima M Kanaide H Dissociation between the Ca (2+) signal and tube formation induced by vascular endothelial growth factor in bovine aortic endothelial cells Eur J Pharmacol 2000 398 19 29 10.1016/S0014-2999(00)00296-X 10856444

[B43] Margheri F Serrati S Lapucci A Anastasia C Giusti B Pucci M Torre E Bianchini F Calorini L Albini A Ventura A Fibbi G Del Rosso M Systemic sclerosis-endothelial cell antiangiogenic pentraxin 3 and matrix metalloprotease 12 control human breast cancer tumor vascularization and development in mice Neoplasia 2009 11 1106 1115 19794969 10.1593/neo.09934 PMC2745676

[B44] Patterson C Perrella MA Hsieh CM Yoshizumi M Lee ME Haber E Cloning and functional analysis of the promoter for KDR/flk-1, a receptor for vascular endothelial growth factor J Biol Chem 1995 270 23111 23118 10.1074/jbc.270.39.23111 7559454

